# Quercitrin Stimulates Hair Growth with Enhanced Expression of Growth Factors via Activation of MAPK/CREB Signaling Pathway

**DOI:** 10.3390/molecules25174004

**Published:** 2020-09-02

**Authors:** Jaeyoon Kim, Soon Re Kim, Yun-Ho Choi, Jae young Shin, Chang Deok Kim, Nae-Gyu Kang, Byung Cheol Park, Sanghwa Lee

**Affiliations:** 1 LG Household & Health Care (LG H&H) R&D Center, 70, Magokjoongang 10-ro, Gangseo-gu, Seoul 07795, Korea; kjy5281@lghnh.com (J.K.); youknow@lghnh.com (Y.-H.C.); sjy2811@lghnh.com (J.y.S.); ngkang@lghnh.com (N.-G.K.); 2 Department of Dermatology, School of Medicine, Chungnam National University, 266, Munwha-ro, Jung-gu, Deajeon 35015, Korea; cdkimd@cnu.ac.kr; 3 Basic and clinical Hair institute, Dankook University, 201, Manghyang-ro, Dongnam-gu, Cheonan-si, Chungcheongnam-do 31116, Korea; sl715@nate.com (S.R.K.); 4exodus@dankook.ac.kr (B.C.P.); 4 Department of Dermatology, Dankook University Hospital, 201, Manghyang-ro, Dongnam-gu, Cheonan-si, Chungcheongnam-do 31116, Korea

**Keywords:** quercitrin, human DPCs, hair growth, growth factors, MAPK, CREB

## Abstract

The present study aimed to investigate the molecular mechanism of quercitrin, a major constituent of *Hottuynia cordata* extract, for its hair growth stimulating activities in cultured human dermal papilla cells (hDPCs). Quercitrin enhanced the cell viability and cellular energy metabolism in cultured hDPCs by stimulating the production of NAD(P)H and mitochondrial membrane potential (ΔΨ). The expression of Bcl2, an essential marker for anagen hair follicle and cell survival, was increased by quercitrin treatment. Quercitrin also increased the cell proliferation marker Ki67. The expression of growth factors—such as bFGF, KGF, PDGF-AA, and VEGF—were increased by quercitrin both in mRNA and protein levels. In addition, quercitrin was found to increase the phosphorylation of Akt, Erk, and CREB in cultured hDPCs, while inhibitors of MAPKs reversed the effects of quercitrin. Finally, quercitrin stimulated hair shaft growth in cultured human hair follicles. Our data obtained from present study are in line with those previously reported and demonstrate that quercitrin is (one of) the active compound(s) of *Hottuynia cordata* extract which showed hair growth promoting effects. It is strongly suggested that the hair growth stimulating activity of quercitrin was exerted by enhancing the cellular energy metabolism, increasing the production of growth factors via activation of MAPK/CREB signaling pathway.

## 1. Introduction

Androgenetic alopecia (AGA) is the most common type of hair loss in men and is a naturally progressive disorder [1]. In the process of AGA, large terminal scalp hairs are gradually replaced by thinner and smaller vellus hairs, which is called hair follicle miniaturization. The pathogenesis and underlying molecular mechanisms of AGA are not yet fully understood but androgen hormone, dihydrotestosterone (DHT) is suspected to be the most potent causative agent in genetically predisposed individuals [2].

Although AGA is not a life-threatening disease it has great psychosocial impact on affected patients, resulting in a significant impairment of quality of life [3]. Despite tremendous efforts for curing AGA, just two drugs—minoxidil and finasteride—were approved by the US Food and Drug Administration (FDA). Among those, minoxidil is the only topical treatment for AGA but the side effects such as pruritus, dermatitis, and irritation have been reported [4].

The hair follicle undergoes three cyclic stages of growth; anagen (proliferation), catagen (involution), and telogen (resting) [5]. Dermal papilla cells (DPCs), differentiated from mesenchymal stem cells and located in the core of hair follicle, are one of the major regulators of hair cycle which controls external stimuli and signals delivered through cytokines and junctions [6]. In AGA, repeated hair cycles with shortened anagen result in hair follicle miniaturization, characterized by depletion of anagen follicles and presence of vellus hairs on affected scalp [7]. Mesenchymal stem cell-derived signals and growth factors from DPCs could influence hair growth through proliferation of hair follicle cells. They could prolong the anagen (KGF), stimulate hair follicle development (β-catenin), or suppress apoptotic cues (Bcl-2) [8]. Stimulating the production of growth factors from DPCs such as bFGF, KGF, PDGF-AA, and VEGF could be effective therapeutic candidates for activating hair cycle and growth.

We have previously reported that the extract of *Houttuynia cordata* (HCE) promoted hair growth in cultured hDPCs and human hair follicle culture model, conferred by elongation of hair follicle anagen [9]. The HCE may contain thousands of chemicals but we have found the presence of four compounds—chlorogenic acid, rutin, quercitrin, and quercetin—as major constituents. Among these, quercitrin was the most abundant one with concentration of approximately 16 mg/mL (1.6%). We have postulated that most (if not all) of the hair growth promoting activity of HCE had come from quercitrin.

Quercitrin (quercetin-3-*O*-rhamnoside) is a natural flavonoid widely found in the flowers, leaves, and fruits of various plants and was reported as anti-inflammatory [10] and anti-oxidant agents [11]. However, the effects of quercitrin on hair growth and alopecia treatment, as we know, were not reported.

In this study, physiological effects of quercitrin on cultured human DPCs and its molecular mechanisms were investigated to prove our hypothesis in which quercitrin is the active ingredient of HCE in stimulation of hair growth.

As expected, quercitrin was found to stimulate the mitochondrial energy metabolism and enhance proliferative capacity, accompanied by increased production of growth factors like bFGF, KGF, PDGF-AA, and VEGF, essential for hair growth. Furthermore, the expression of Bcl2 and Ki67, typical markers for cell survival and anagen extension, was also increased. The cell signal transduction elements—such as Akt, Erk, CREB and several receptor tyrosine kinases—were found to be stimulated by quercitrin. In addition, quercitrin enhanced the hair shaft growth in cultured human hair follicles. Our data strongly suggest that the hair growth stimulating activity of HCE comes mainly from quercitrin and that quercitrin possesses possible therapeutic potential for preventing and/or treating the hair loss.

## 2. Results

### 2.1. Quercitrin Increased Cell Viability in Cultured Human DPCs

**Figure 1:**
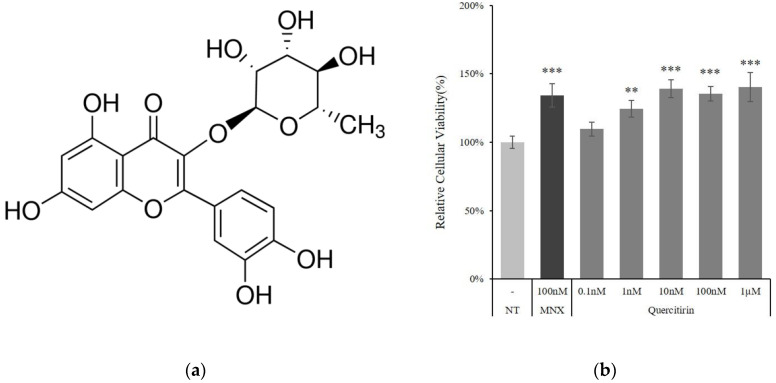
Quercitrin enhanced cell viability in cultured hDPCs. (**a**) Quercitrin chemical structure. (**b**) Cell viability was assessed using CCK-8 assay kit after quercitrin treatment (0.1, 1, 10, 100 nM and 1 µM) for 24 h. The value of non-treated control was taken to be 100%. N.T, non-treated control; MNX, minoxidil. Significantly different compared with N.T (* *p* < 0.05, ** *p* < 0.01, *** *p* < 0.001).

The treatment of quercitrin increased the viability of DPCs in a concentration dependent manner. NAD(P)H generation, presented as cell viability, was increased by 9.6%, 24.4%, and 39.0% in the presence of 0.1, 1, and 10 nM quercitrin, respectively. Above 10 nM concentrations, the cell viability was reached to a plateau (Figure 1b). The increments were comparable with that of 100 nM of minoxidil, approved topical hair growth stimulating medicine, a positive control.

**Figure 2:**
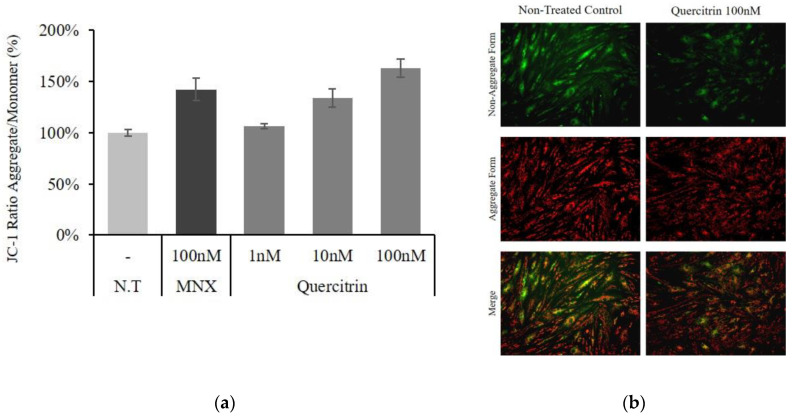
Effect of quercitrin on cellular energy metabolism in cultured hDPCs. (**a**) Mitochondrial membrane potential was measured after quercitrin treatment (1, 10, and 100 nM) for 24 h. The 100 nM minoxidil was used as a positive control. (**b**) JC-1 monomer form was seen as green and aggregate form as red by fluorescent microscopy. N.T, non-treated control; MNX, minoxidil. Significantly different compared with N.T (* *p* < 0.05, ** *p* < 0.01, *** *p* < 0.001).

Because NAD(P)H production was markedly increased by quercitrin treatment, the changes in mitochondrial membrane potential (∆Ψ) were investigated. The mitochondrial membrane potential indicates the capacity for energy generation in mitochondria. As shown in Figure 2, the membrane potential was increased in a concentration dependent manner. The treatment of quercitrin at concentration of 10 and 100 nM significantly increased the membrane potential by 34.0% and 63.2%, respectively. Combining the data in Figure 1 and Figure 2, the treatment of quercitrin resulted in potentiation of energy metabolism in cultured hDPCs.

### 2.2. Quercitrin Enhanced Bcl2 Expression

**Figure 3:**
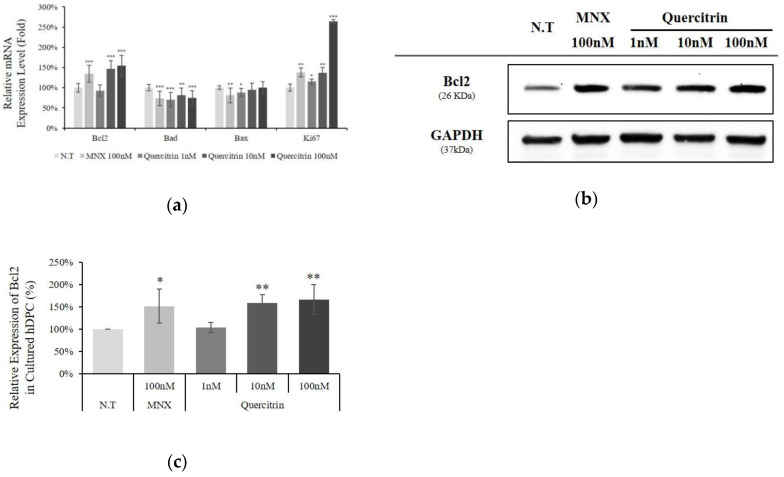
Effect of quercitrin on mRNA expression levels of proliferative/apoptotic genes and protein level of Bcl2 in cultured hDPCs. The cultured DPCs were harvested after quercitrin treatment (1, 10, and 100 nM) for 24 h. (**a**) The mRNA expression levels of Bcl2, Bad, Bax and Ki67 genes in cultured hDPCs were measured by real-time PCR. (**b**) Whole cell lysates (50 µg protein) from DPCs were analyzed by immunoblotting to determine the levels of Bcl2, and (**c**) the band intensity was quantitated. N.T, non-treated control; MNX, minoxidil. Significantly different compared with N.T (* *p* < 0.05, ** *p* < 0.01, *** *p* < 0.001).

The mRNA expression level of cell viability related genes was assessed in cultured hDPCs. The expression of Bad, an apoptotic marker in mitochondria, was significantly decreased by quercitrin treatment. On the contrary, an anti-apoptotic gene Bcl2 level was significantly increased by 47% and 54% with 10 and 100 nM quercitrin, respectively. In addition, cell proliferation marker, Ki67 was also increased by quercitrin in a concentration dependent manner (Figure 3a). Western blot analysis revealed that quercitrin also significantly increased the amount of Bcl2 protein by 66.2% with 100 nM treatment (Figure 3b,c).

### 2.3. Quercitrin Increased mRNA Expression of Growth Factors and Their Receptors

**Figure 4:**
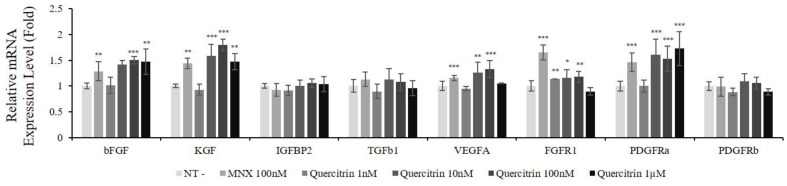
Effect of quercitrin on mRNA expression levels of growth factor genes in cultured hDPCs. The cells were harvested after quercitrin treatment (1, 10, 100 nM and 1 µM) for 24 h. The mRNA expression levels of eight genes in cultured hDPCs were measured by real-time PCR. N.T, non-treated control; MNX, minoxidil. Significantly different compared with N.T (* *p* < 0.05, ** *p* < 0.01, *** *p* < 0.001).

The DPCs secrete myriad of growth factors to regulate hair follicle growth. To specify the factors affecting metabolic stimulation and cell viability, the mRNA expression of growth factors and growth factor receptors was evaluated. The expression levels of 19 growth factor genes and 15 growth factor receptor genes were measured. Among 34 genes tested, the expression of 8 genes (bFGF, KGF, IGFBP2, TGFb1, VEGFA, FGFR1, PDGFRa, and PDGFRb) was increased by quercitrin treatment. Among growth factors, mRNA expression of bFGF, KGF and VEGFA was significantly increased by 50.1%, 79.5%, and 32.7% at 100 nM quercitrin treatment, respectively. For growth factor receptors, the expression level of FGFR1 and PDGFRa was significantly increased (Figure 4).

### 2.4. Quercitrin Increased the Protein Expression of Growth Factors

**Figure 5:**
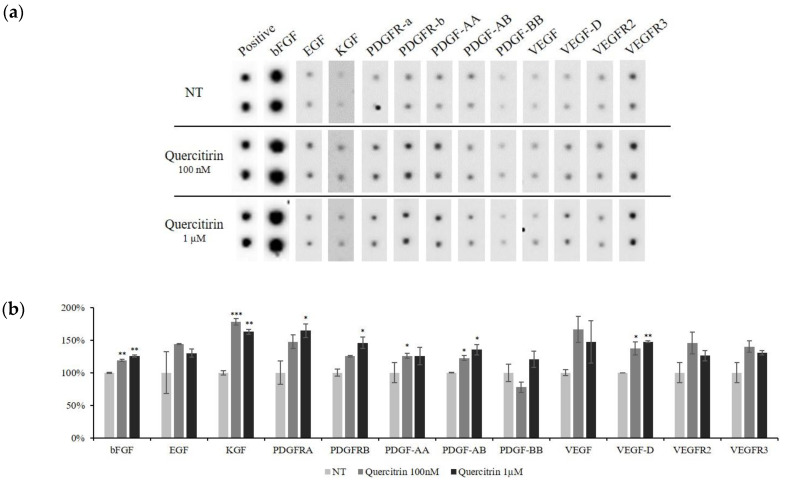
Effect of quercitrin on the expression of growth factors in cultured hDPCs. The DPCs were treated with quercitrin (0, 100 nM and 1 µM) for 24 h, and then collected. Cells cultured with vehicle medium were used as non-treated control. Total of 41 types of human growth factors were analyzed. (**a**) The 12 types of growth factors and receptors were displayed and (**b**) the band intensity was quantitated. N.T, non-treated control. Positive, biotin-conjugated IgG. Significantly different compared with N.T (* *p* < 0.05, ** *p* < 0.01, *** *p* < 0.001).

To elucidate whether quercitrin affect the gene expression of growth factors and their receptors to protein level, dot blot analysis was performed. The amount of bFGF, KGF, PDGF-AA, and VEGF, which were reported to enhance the cell viability of DPCs [12], was significantly increased by quercitrin treatment (Figure 5).

**Figure 6:**
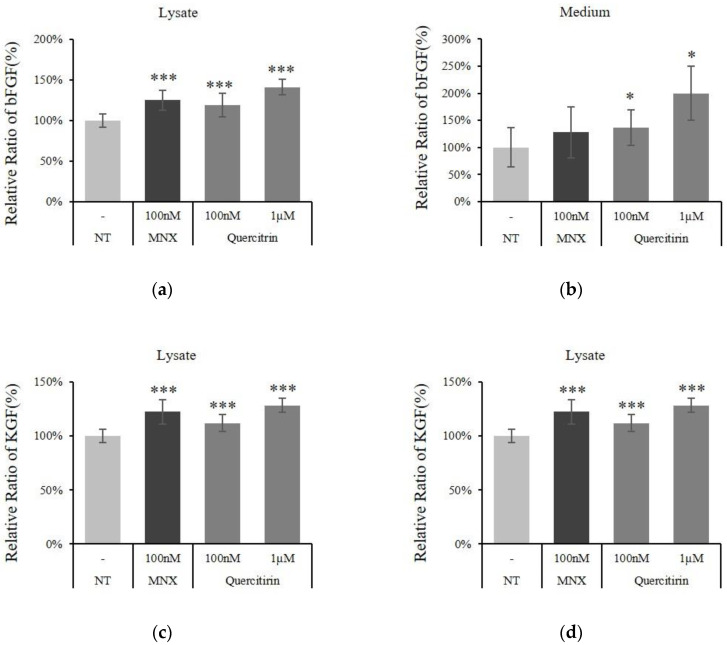
Effect of quercitrin on the protein expression of bFGF and KGF in cultured hDPCs. The DPCs were treated with quercitrin at concentrations of 1, 10, 100 nM and 1 µM for 24 h. Whole cell lysates (**a**,**c**) and culture medium (**b**,**d**) of each cultured DPCs were analyzed by ELISA to determine the levels of bFGF (a,b) and KGF (c,d). N.T, non-treated control. Significantly different compared with N.T (* *p* < 0.05, ** *p* < 0.01, *** *p* < 0.001).

Protein levels of bFGF and KGF both in culture medium and cell lysate were further investigated using ELISA to differentially assess the intracellular and secreted form. The intracellular bFGF and KGF were significantly increased by quercitrin treatment (Figure 6a,c). Furthermore, quercitrin at concentration of 100 nM also increased the secretion of bFGF and KGF by 36.5% and 27.3%, respectively (Figure 6b,d). Taken together, quercitrin enhanced not only the production of bFGF and KGF but also the secretion of them.

### 2.5. Quercitrin Induced Phosphorylation of Akt, Erk, and CREB

**Figure 7:**
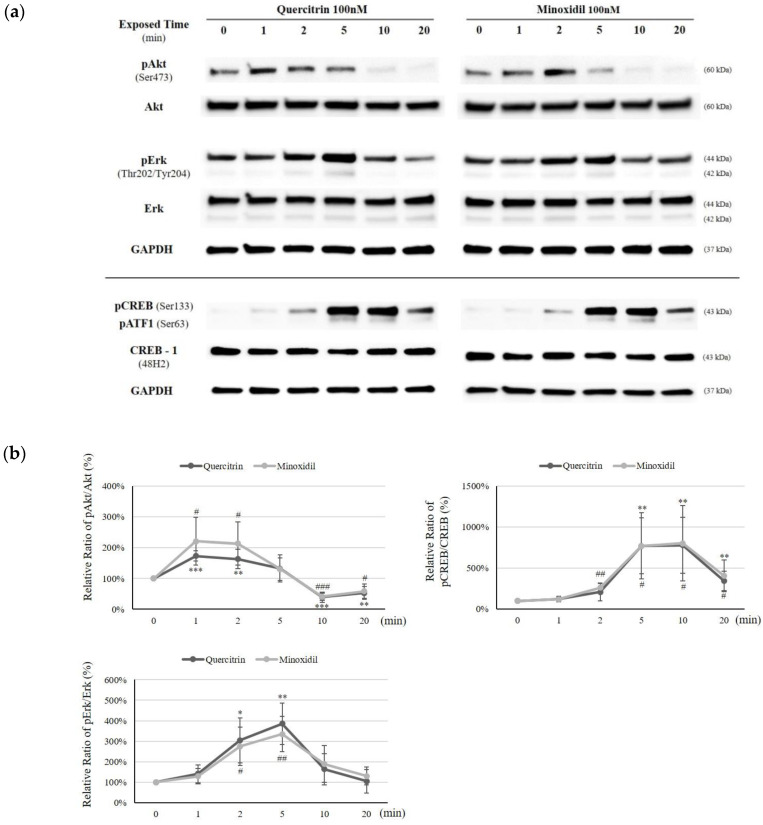
Effect of quercitrin on Akt, Erk, and CREB phosphorylation in cultured hDPCs. Quercitrin was treated for different times (0, 1, 2, 5, 10, and 20 min). (**a**) Whole cell lysates were analyzed by immunoblotting to determine the levels of Akt, phospho-Akt, Erk, phospho-Erk, CREB and phospho-CREB. As an internal control, GAPDH were used. (**b**) The ratio of pAkt/Akt, pErk/Erk, and pCREB/CREB was calculated. N.T, non-treated control. The data represent the means of five independent samples. Significantly different compared with N.T (Quercitrin * *p* < 0.05, ** *p* < 0.01, *** *p* < 0.001; Minoxidil # *p* < 0.05, ## *p* < 0.01, ### *p* < 0.001).

To clarify the molecular action mechanism of quercitrin, the MAP kinase molecules, which play pivotal roles in cellular signal cascades, were investigated. Among phosphorylated MAP kinase proteins, phospho-Akt and phospho-Erk were significantly increased. The protein levels of total Akt and Erk, on the other hand, were not changed. It was revealed that the phosphorylation of Akt and Erk tended to have a time dependency with maximum increases at a certain time point (Figure 7a). The ratio of pAkt/Akt was increased by 73.3% and 63.8%, when 100 nM of quercitrin was treated for 1 and 2 min, respectively. On the other hand, pErk/Erk ratio was more prominently increased by 204.0% and 285.9%, for 2 and 5 min of quercitrin treatment, respectively (Figure 7b). As a downstream target of Thr202-Erk phosphorylation, CREB phosphorylation at Ser133 site was examined [13]. The phospho (Ser133)-CREB/CREB level was significantly elevated by 671.4%, 680.0%, and 244.0%, when 100 nM of quercitrin was treated for 5, 10, and 20 min, respectively (Figure 7b).

**Figure 8:**
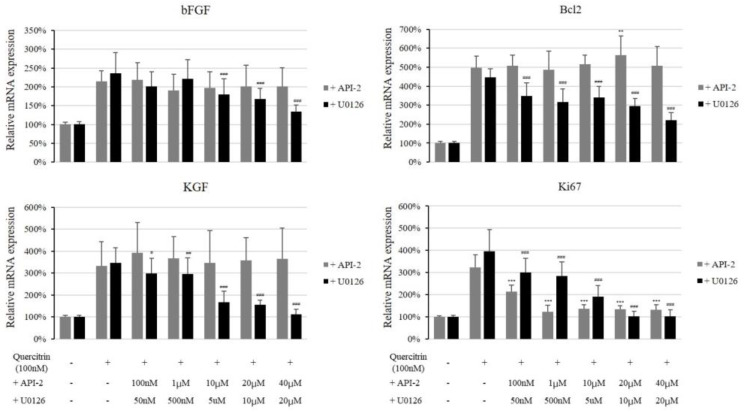
Effect of Akt inhibitor API-2 and Erk inhibitor U0126 on quercitrin stimulated gene expression. The cultured DPCs were harvested after 100 nM quercitrin treatment with inhibitors (API-2 and U0125) for 24 h. The mRNA expression levels of bFGF, KGF, Bcl2, and Ki67 were measured by real-time PCR. The data represent the means of six independent samples. Significantly different compared with 100 nM quercitrin treatment (API-2 * *p* < 0.05, ** *p* < 0.01, *** *p* < 0.001; U0126 # *p* < 0.05, ## *p* < 0.01, ### *p* < 0.001).

We scrutinized whether the inhibitors of MAP kinases could inhibit the quercitrin induced gene expressions to find out the relationship between enhanced gene expression and MAPKs. As shown in Figure 8, Erk inhibitor U0126 (20 µM) significantly diminished the quercitrin stimulated expression of bFGF, KGF and Bcl2 while Akt inhibitor API-2 did not. The quercitrin-induced Ki67 expression, on the other hand, was inhibited by both U0125 and API-2. Our data suggest that quercitrin-stimulated expression of growth factors (bFGF and KGF) and Bcl2 was mediated by Erk activity and that both Erk and Akt were involved in enhanced cell proliferative potential by quercitrin.

### 2.6. Quercitrin Activated Receptor Tyrosine Kinases and Non-Receptor Tyrosine Kinases

**Figure 9:**
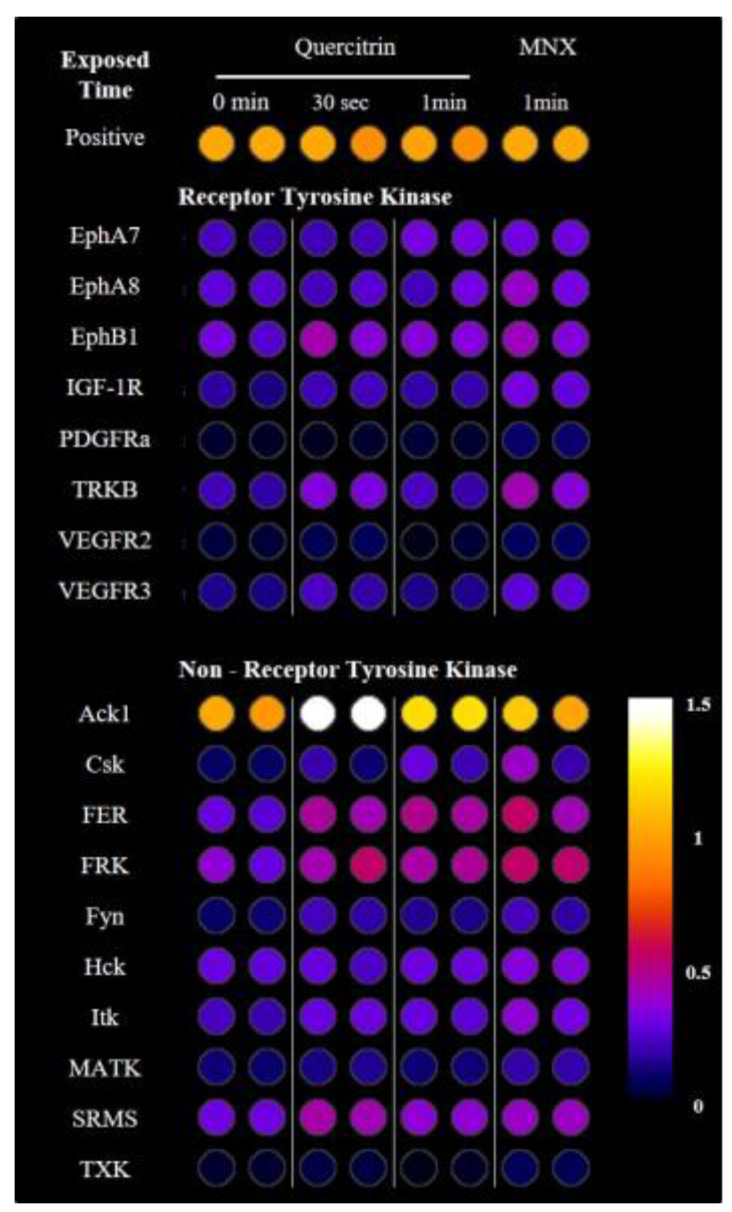
Quercitrin stimulated the receptor tyrosine kinases and non-receptor tyrosine kinases in cultured hDPCs. 100 nM of quercitrin was treated for appropriate times (0, 0.5, 1 min). Whole cell lysates were analyzed by immunoblotting to determine the level of phospho-tyrosine following manufacturer’s instruction. Total of 71 types of tyrosine kinase were analyzed. The 8 receptor tyrosine kinases and 10 non-receptors tyrosine kinases were displayed (Significantly different compared with 0 min, *p* < 0.05). Positive, biotin-conjugated IgG.

The receptor tyrosine kinase and its receptor activation pathways were well proved for the activation of Akt and Erk/MAPK pathways [14]. Therefore, we investigated the phosphorylation of tyrosine kinases using RTK dot blotting in cultured DPCs treated with quercitrin for 30 s and 1 min. Total of 71 phosphorylated proteins were examined and 18 proteins were detected with significant increase by quercitrin treatment (*p* < 0.05), 8 receptor tyrosine kinases and 10 non-receptor tyrosine kinases including Src family proteins (Figure 9).

### 2.7. Quercitrin Stimulated Hair Growth Ex Vivo

**Figure 10:**
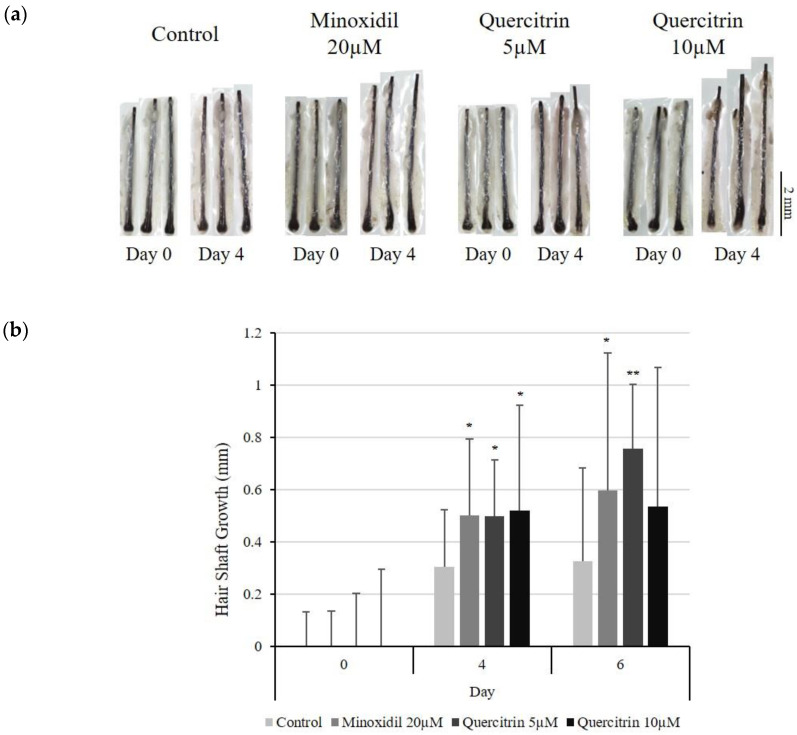
Effect of quercitrin on hair growth in human hair follicle organ culture. In order to evaluate the effect of quercitrin, the anagen human hair follicle were prepared and cultured for 6 days. Quercitrin was treated at concentrations of 5, 10 µM. (**a**) At day 4 and 6, the cultured hair follicles were photo-documented. (**b**) The hair shaft growth was analyzed. Minoxidil was used as a positive control. The data represent the means of sixteen follicles. Significantly different compared with N.T (* *p* < 0.05, ** *p* < 0.01, *** *p* < 0.001).

The effect of quercitrin on human hair growth was investigated in human hair follicle organ culture model. Quercitrin significantly increased the hair growth compared with non-treated control (Figure 10). On day 4 of culture, quercitrin increased the length of hair shaft by 62.4% (5 µM) and 70.0% (10 µM) compared with non-treated control. A positive control minoxidil showed a comparable result.

## 3. Discussion

The hair follicle is one of the most energy-consuming organs in humans so energy-genesis processes are highly activated in human hair follicle cells. In DPCs, mitochondria strongly contribute to energy generation [15], and the enhancement of mitochondrial function affects hair growth in vitro and in vivo [16]. Therefore, mitochondria and their energy production are regarded as important for promoting hair growth.

The mitochondrial energy generation is determined by mitochondrial membrane potential (ΔΨ), which in turn is tightly conjugated with positive steady state of NAD/NADH coupling. Quercitrin treatment was found to increase not only NADH production, but also mitochondrial membrane potential (Figure 1b and Figure 2). In this context, our data strongly suggest that quercitrin could stimulate mitochondrial activities in human DPCs, which might be related to hair follicle morphogenesis [17].

The Bcl-2 family proteins were shown to manage cell senescence and administer cellular apoptosis [18]. Among the family members, Bcl2 in particular protects cells from stress challenges [19] and higher Bcl2 level prevents apoptosis [20]. In hair follicles, interestingly, the Bcl2 level is significantly higher in DPCs than other cell types [21]. In addition, high level of Bcl2 is essential for maintaining anagen and keeping DPCs function properly [22]. As mentioned above, Bcl2 level was increased and Bad level was decreased by quercitrin treatment (Figure 3). Our data demonstrate the possibility that quercitrin could maintain the hair follicle in anagen state.

In hair follicle, growth factors—such as bFGF, KGF, PDGF-AA, and VEGF—were reported to activate hair cycle and growth [8] and to be secreted by DPCs. Especially, KGF is one of the DPCs’ signal that participates in instructing hair germ to proliferate and induce a new hair cycle [23]. In the present study, quercitrin was found to stimulate the production of these growth factors in cultured hDPCs (Figure 4, Figure 5 and Figure 6). It could be predicted that the growth factors contributing to prolong the anagen and support hair growth could be supplied to hair bulb in higher level by quercitrin treatment.

As previously described, quercitrin activated Akt and Erk/CREB signaling in DPCs. The activation of CREB is strongly conjugated with mitochondrial activation, facilitating mitochondrial gene expression and cell survival through phosphor-CREB (Ser133) binding to CRE site in the mitochondrial D-loop DNA [24]. The cellular improvements are accompanied by corresponding expression of CREB-targeted genes, such as Bcl2 (cell survival) and PGC-1α (mitochondrial biogenesis) [25]. These findings are consistent with our data that quercitrin stimulated Bcl-2 expression and mitochondrial function via activation of CREB.

Furthermore, many growth factors were reported to be regulated by MAPK/CREB signaling pathway. VEGF expression is regulated by PI3K/Akt-NF-κB cascade [26] or MAPK/ERK pathway [27]. The promoter region of the KGF gene has transcriptional regulatory element for activating transcription factor (ATF) family of CREB proteins [28], and CREB activation stimulate KGF gene expression in DPCs [29]. bFGF and PDGF-AA genes are also the target of early growth response gene-1 (EGR1) which is activated by Erk phosphorylation [30].

The receptor tyrosine kinase or related non-receptor tyrosine kinases, for example Src family proteins, are possibly involved in the activation of Akt and Erk pathways and the regulation of PI3K and MAPK pathways by Src family proteins are well-established [31]. As shown in Figure 9, several receptor tyrosine kinases and non-receptor tyrosine kinases were activated by quercitrin treatment. Among those, Src family proteins—such as Csk, FRK, Hck, and SRMS—are of special interest because they, as non-receptor tyrosine kinase, not only interact with the protein-tyrosine kinase receptors at cell membrane but also participate in numerous signaling pathways. Further studies for unveiling the role of Src family proteins in the activation of DPCs by quercitrin would be challenging.

In this report, we have found that quercitrin activated the energy metabolism and enhanced the production of growth factors including bFGF and KGF. We also found that Bcl2 and Ki67 levels were upregulated and Bad level was downregulated. It seems plausible that all these stimulations are related to the activation of Akt, Erk, and CREB signal transduction pathways (Figure 7). The Erk inhibitor U0126 but not Akt inhibitor API-2 was found to inhibit the quercitrin-induced expression of growth factors and Bcl2, strongly implying the involvement of Erk activation in its mechanism of action. The cell proliferation marker Ki67 was decreased by both Erk inhibitor U0126 and Akt inhibitor API-2. In addition, several receptor tyrosine kinases and their conjugated tyrosine kinases were also activated by quercitrin as an upstream of Akt and Erk/CREB signaling (Figure 9). Finally, quercitrin stimulated hair growth in a human hair follicle culture model, confirming our hypothesis.

We could not exclude, however, the possibility that the biological activities including hair growth promoting activity came from other constituents or unidentified ones of the HCE even though they took a little since other major components like chlorogenic acid and quercetin showed stimulatory activities in CCK-8 assay. Although quercitrin was our first choice for further investigation because of its abundance, investigating the roles of other major components of the HCE on hair growth might be interesting.

In conclusion, our data strongly suggest that quercitrin is the major active component of HCE and stimulates hair growth by enhancing the cellular energy metabolism and increasing the secretion of growth factors through the activation of MAPK/CREB signaling pathway, demonstrating the possible therapeutic potential of quercitrin for treating and/or preventing hair loss.

## 4. Materials and Methods

### 4.1. Dermal Papilla Cells Culture

Human DPCs were purchased from Promocell (Heidelberg, Germany). DPCs were cultured in basal medium supplemented with 4% fetal calf serum, 0.4% bovine pituitary extract, 1 ng/mL basic fibroblast growth factor and 5 μg/mL insulin (Supplement Mix, Promocell). Cells were maintained in humidified incubator at 37 °C, 5% CO_2_. Before quercitrin (Sigma-Aldrich, MO, USA; Figure 1a) treatment, serum limitation was done by replacing the medium with fresh DMEM (Thermofisher scientific, Waltham, MA, USA) supplemented with 1% FBS (Thermofisher scientific, Waltham, MA, USA) and 1 ng/mL bFGF (Merck, Darmstadt, Germany) and culturing for 24 h to minimize the effects of serum and growth supplements. Akt inhibitor API-2 and Erk inhibitor U0126 were purchased from Tocris Bioscience (Bristol, UK).

### 4.2. Cell Viability Assay

The effect of quercitrin on the viability of DPCs was examined using CCK-8 assay (Dojindo, MA, USA) and JC-1 mitochondrial membrane potential assay (Abcam, Cambridge, UK) kits following the manufacturer’s protocols. For examining the cellular energy metabolism, NAD(P)H generation was measured by CCK-8 assay. The absorbance at 450 nm was read using micro-plate reader (BioTek, Winooski, VT, USA). The mitochondrial membrane potential was measured by JC-1 staining. Briefly, after quercitrin treated DPCs were stained with 1 μM JC-1 solution, fluorescence intensities from JC-1 aggregate and monomer form were measured at 590 nm (535 nm excitation) and 530 nm (475 nm excitation), respectively, with Wallac Victor3 1420 (PerkinElmer, Waltham, MA, USA). Mitochondrial membrane potential (∆Ψ) was visualized by taking fluorescence images with EVOS™ FL Auto2 Imaging System (Thermofisher scientific, Waltham, MA, USA).

### 4.3. Quantitative Real-Time PCR

Quercitrin was treated at concentrations of 1, 10, 100 nM and 1 μM for 24 h, with non-treated cells served as control. Total RNA was extracted using Rneasy RNA extraction kit (Qiagen Inc., CA, Germantown, USA). cDNA synthesis was performed using cDNA synthesis kit (Phillkorea, Seoul, Korea) with ThermoCycler (R&D systems, Minneapolis, MN, USA), according to the manufacturer’s protocol. cDNA samples obtained from control and treated cells were subjected to real-time(RT) PCR analysis.

TaqMan probes for RT-PCR used in this study were as follows: GAPDH assay id 4352934E; Bcl2 assay id Hs00608023_m1; BAD assay id Hs00188930_m1; Bax assay id Hs00180269_m1; MKI67 assay id Hs04260396_g1; bFGF (FGF2) assay id Hs00266645_m1; KGF (FGF7) assay id Hs00940253_m1; IGFBP2 assay id Hs01040719_m1; TGFb1 assay id Hs00998133_m1; VEGFA assay id Hs00900055_m1; FGFR1 assay id Hs00241111_m1; PDGFRa assay id Hs01019589_m1; PDGFRb assay id Hs00998018_m1.

TaqMan One-Step RT-PCR Master Mix Reagent (Life Technologies, Carlsbad, CA, USA) was used. The PCR reactions were performed on ABI 7500 Real Time PCR system following the manufacturer’s instruction. The resulting data were analyzed with ABI software (version).

### 4.4. Western Blot Analysis

DPCs (1 × 10 ^6^ cells/dish) were seeded in 100 mm dishes and cultured for 24 h. Quercitrin was treated at concentrations of 1, 10, and 100 nM for appropriate time. The cells were then lysed and total cellular proteins were prepared. 50 μg protein samples were analyzed by Western blotting with corresponding antibodies; Bcl2 (1:1000, Abcam, cambridge, UK), Akt (1:1000, Santa Cruz, CA, USA), Erk (p44/42) (1:1000, Cell Signaling Technology, Danvers, MA, USA), CREB-1 (Cell Signaling Technology, Danvers, MA, USA) GAPDH (1:2000, Santa Cruz, CA, USA), phospho (Ser473)-Akt (1:1000, Cell Signaling Technology, Danvers, MA, USA), phospho (Thr202/Tyr204)-Erk (p44/42) (1:1000, Cell Signaling Technology, Danvers, MA, USA), and phospho (Ser133)-CREB (1:1000, Cell Signaling Technology, Danvers, MA, USA). Western blot was analyzed by chemiluminescence detector iBright FL1000 (Invitrogen, Waltham, MA, USA).

### 4.5. Protein Dot Blot Analysis for Growth Factors (Receptors) and Receptor Tyrosine Kinase Phosphorylation

Human growth factor antibody array kit (Abcam, Cambridge, UK) and human receptor tyrosine kinase (RTK) phosphorylation antibody array kit (Abcam, Cambridge, UK) were used to elucidate the changes in growth factor profiles and signal transduction pathways in DPCs. Total of 41 human growth factors and 71 human RTK phosphorylation were analyzed. Cells were treated with 100 nM and 1 µM of quercitrin for appropriate time and then collected for growth factor and RTK phosphorylation analysis. Cells treated with vehicle medium were used as non-treated control. Conventional immunoblot process was performed following the manufacturer’s protocol. The resulting blots were analyzed under identical condition using iBright FL1000 (Invitrogen, Waltham, MA, USA).

### 4.6. bFGF and KGF ELISA

DPCs were treated with various concentrations of quercitrin for 24 h. The cells and culture medium were collected and the amounts of bFGF and KGF were measured using human bFGF and human KGF DuoSet ELISA (R&D systems) kits, according to the manufacturer’s instruction. Absorbance at 450 nm was measured using microplate reader (BioTek, Winooski, VT, USA). Background wavelength correction was done at 540 nm.

### 4.7. Human Hair Follicle Organ Culture

Human scalp skin was obtained from nonbalding areas from patients undergoing hair transplant surgery with written consent and approval by the Institutional Review Board of Dankoon University Hospital (IRB no. DKUH. 2017-07-003). Human hair follicles were isolated by microdissection under the microscope. Anagen VI hair follicles were chosen for the study. Isolated hair follicles were maintained in William’s E medium (Life Technologies, Carlsbad, CA, USA) supplemented with 10 μg/mL insulin (Sigma-Aldrich, St. Louis, MO, USA), 10  ng/mL hydrocortisone (Sigma-Aldrich, St. Louis, MO, USA), 2 mM L-glutamine (Life Technologies, Carlsbad, CA, USA), and 10 U/mL penicillin (Life Technologies, Carlsbad, CA, USA), 100 ug/mL streptomycin (Life Technologies, Carlsbad, CA, USA), and 25 ug/mL amphotericin B (Life Technologies, Carlsbad, CA, USA). All cultures were incubated at 37 °C in an atmosphere of 5% CO_2_ and 95% air.

### 4.8. Statistical Analysis

All experimental data were presented as the mean ± standard deviation (S.D.) of at least three independent experiments. Experimental results were analyzed using the SigmaPlot 8.0 (Systat Software Inc., Chicago, IL, USA). The statistical significance of difference was determined by Student’s *t*-test. The value of *p* < 0.05 considered statistically significant.
